# Compression, expansion, or maybe both? Growing inequalities in lung cancer in Germany

**DOI:** 10.1371/journal.pone.0242433

**Published:** 2020-11-20

**Authors:** Fabian Tetzlaff, Jelena Epping, Heiko Golpon, Juliane Tetzlaff

**Affiliations:** 1 Institute for General Practice, Hannover Medical School, Hanover, Germany; 2 Medical Sociology Unit, Hannover Medical School, Hanover, Germany; 3 Comprehensive Cancer Center Hannover, Hannover Medical School, Hanover, Germany; 4 Department of Pneumology, Hannover Medical School, Hanover, Germany; Sciensano, BELGIUM

## Abstract

**Background:**

Lung Cancer (LC) is one of the most common malign diseases worldwide. So far, it is unclear if the development of LC incidence and mortality leads to morbidity compression or expansion and whether these developments differ by socioeconomic characteristics. This study analyses time trends in social and gender inequalities in life years with and without LC in Germany.

**Methods:**

The study is based on data of a large German statutory health insurance provider (N = 2,511,790). Incidence and mortality risks were estimated from multistate survival models. Trends in life years with and without LC were analysed using multistate life table analyses. All analyses were performed separately for gender, time period (2006–2009 and 2014–2017), and income group (<60% and ≥60% of the German average income).

**Results:**

Among men, declining LC incidence rates resulted in gains of life years free of LC and declining LC- affected life years and led to a relative compression, which was strongest in men with higher incomes. Among women, a clear increase in life years with LC led to an expansion of the lifespan affected by LC. This expansion was mainly driven by increasing incidence rates in women with low incomes. Overall, income inequalities in LC increased in both genders.

**Conclusions:**

Our analyses reveal that developments in the length of life affected by LC differed substantially by gender and income and led to widening health inequalities over time. Public health efforts should mainly focus on vulnerable groups to reduce the persisting social inequalities in LC.

## Introduction

Lung cancer (LC) is one of the most frequent cancer diseases that cause a considerable share of premature deaths globally [1]. While decreasing incidence rates were reported for men, the LC burden in women has increased over the last decades in almost all western countries [2–4]. However, research on social epidemiological trends in LC incidence and mortality is still insufficient and little is known on whether trends in the average lifespan free of LC and after LC incidence differ by socioeconomic characteristics. This holds especially true for the German population, since the official cancer registries do not include information on socioeconomic status (SES) of the diseased individuals.

A wide body of international research has shown the strong link between SES and harmful health-related behaviour and the increased risks of morbidity and mortality (e.g. for Germany [5,6]). This association between SES, harmful health-related behaviour (especially smoking) and increased morbidity risks applies particularly to LC [7–15], causing substantial inequalities in LC incidence. So far, however, there is only a limited number of international studies which investigated time trends in social inequalities in LC morbidity and mortality. The majority of these studies reported growing social inequalities in LC for both genders [10,13–15]. Due to data limitations (especially the lack of sociodemographic information in the official cancer registries), SES inequalities in LC in Germany are still under researched. In a recent study data from German cancer registries were linked with information on the reginal deprivation level. The study reported SES inequalities in LC incidence for men, but not for women [9]. However, studies analysing time trends in inequalities in LC and in the average lifespan free of LC and after morbidity onset are still lacking.

With regard to time trends in the average lifespan spent free of LC and after incidence, different scenarios are possible. Rising incidence rates and increased survival after disease onset may foster prolonged periods spent in ill health and lead to an expansion of morbidity [16]. In contrast, the hypothesis of morbidity compression postulated by Fries [17,18] assumes that efforts in the field of prevention lead to a shift of disease onset to higher ages and extend the period of life spent in good health. With respect to LC, the tabacco control policies established at the end of the last century were the main driving factors in prevention, reduced smoking prevalence and had positive effects on the development of life expectancy, especially in men [19]. However, research suggests that smoking reduced more strongly in individuals with higher education [20–27], which may have increased health inequalities. Furthermore, historical trends in smoking prevalence differ between men and women. While smoking prevalence was already high in men, smoking became more and more common in women in the 1960s and 1970s, which led to a convergence of smoking rates between men and women in many industrialised countries [21,22,27]. Due to the cummulative disadvantaging effect of harmful health-related behaviour, these trends in smoking determine population health in the following decades and may foster social and gender inequalities in health and mortality in later life [22,23,27–35]. Hence, diverging trends in SES-, age- and gender-specific LC incidence may have also caused diverging trends in life years free of LC and those affected by LC.

The aim of the study is to gain a more detailed insight into recent trends in social inequalities in LC incidence and mortality. In particular, the question of compression or expansion of life years affected by LC should be examined in more depth. To analyse these aspects, we used data of a large German statutory health insurance provider. Due to the large number of cases, it was feasible to analyse the development of LC incidence and mortality over time by socioeconomic group and gender and to investigate the resulting trends in life years with and without LC.

This study addresses the following questions:

1. Are there differing time trends in LC incidence and mortality between SES groups? Do these trends differ between men and women?
2. Do time trends in life years free of LC and affected by LC differ by SES group? Do these trends differ between men and women?

## Materials and methods

### Data

As part of the welfare-state system, health insurance is compulsory in Germany. Above a certain income level, inhabitants can choose between statutory health insurance and private health insurance. In total, approximately 90% of the German inhabitants are insured within the statutory health insurance system [36]. Our analyses are based on the data of a large statutory health insurance provider (AOK Niedersachsen [AOKN]) located in the federal state Lower Saxony, which cover the years 2005 to 2017. About 37% of the total population of Lower Saxony is insured with this provider [37]. Our study is based on claims data, i.e., on routinely collected data of a statutory health insurance provider. We confirm that all data are fully anonymised before we accessed them. The use of this sort of data for scientific purposes is regulated by federal law. The data protection officer of the Statutory Local Health Insurance of Lower Saxony (AOK Niedersachsen) has approved its use.

Since the number of LC incident cases in the different subgroups (income, gender, age groups) within one year is limited, time trend analyses are based on comparisons of two time periods (2006–2009 and 2014–2017). The data include detailed sociodemographic (e.g. age, gender, and income) and medical information (e.g. in- and outpatient ICD-10 diagnoses, procedures, and medication) of the insured individuals. Previous analyses have shown that the AOKN population is very similar to the total German population in terms of age and gender distributions, but differs with respect to SES distributions, since individuals without vocational training are overrepresented (approximately +10%) and individuals with university degree are underrepresented (approximately -10%), while the proportion of individuals with vocational training is similar [38]. The analyses focus on individuals aged 40 and older as numbers of incident cases are very limited in younger age groups (0.5% of all LC incidence cases in men and 1.5% in women occur before age 40). The definition of LC incidence is based on the occurrence of an in- or outpatient ICD-10 diagnosis (C34.0 to C34.9) during observation periods. To prevent recurrent diagnoses from being defined as initial diagnoses, pre-observation periods of 90 days were used.

### Socioeconomic characteristics

In this study, trends in SES inequalities in LC incidence and mortality were assessed using information on the annual income of the insured individuals. In Germany, employers are legally bound to report the incomes of their employees to the statutory health insurance providers. Since insurance fees depend on income, self-employed individuals are also obliged to report their incomes. For retired individuals, the income is reported by the Federal Pension Fund. Therefore, the data contain detailed information on annual income, e.g. on salaries and pension payments. The individual income was adjusted for the annual inflation rate to allow direct comparability between the two time periods by keeping the purchasing power constant over time. Finally the study population was classified relative to their income level: The low-income group includes all individuals with an annual income of <60% of the average income from employment in Germany in the respective period as reported by the Federal Statistics Office [39]. Individuals with ≥60% of the average German income were assigned to the higher-income group.

### Statistical analyses

**Fig 1:**
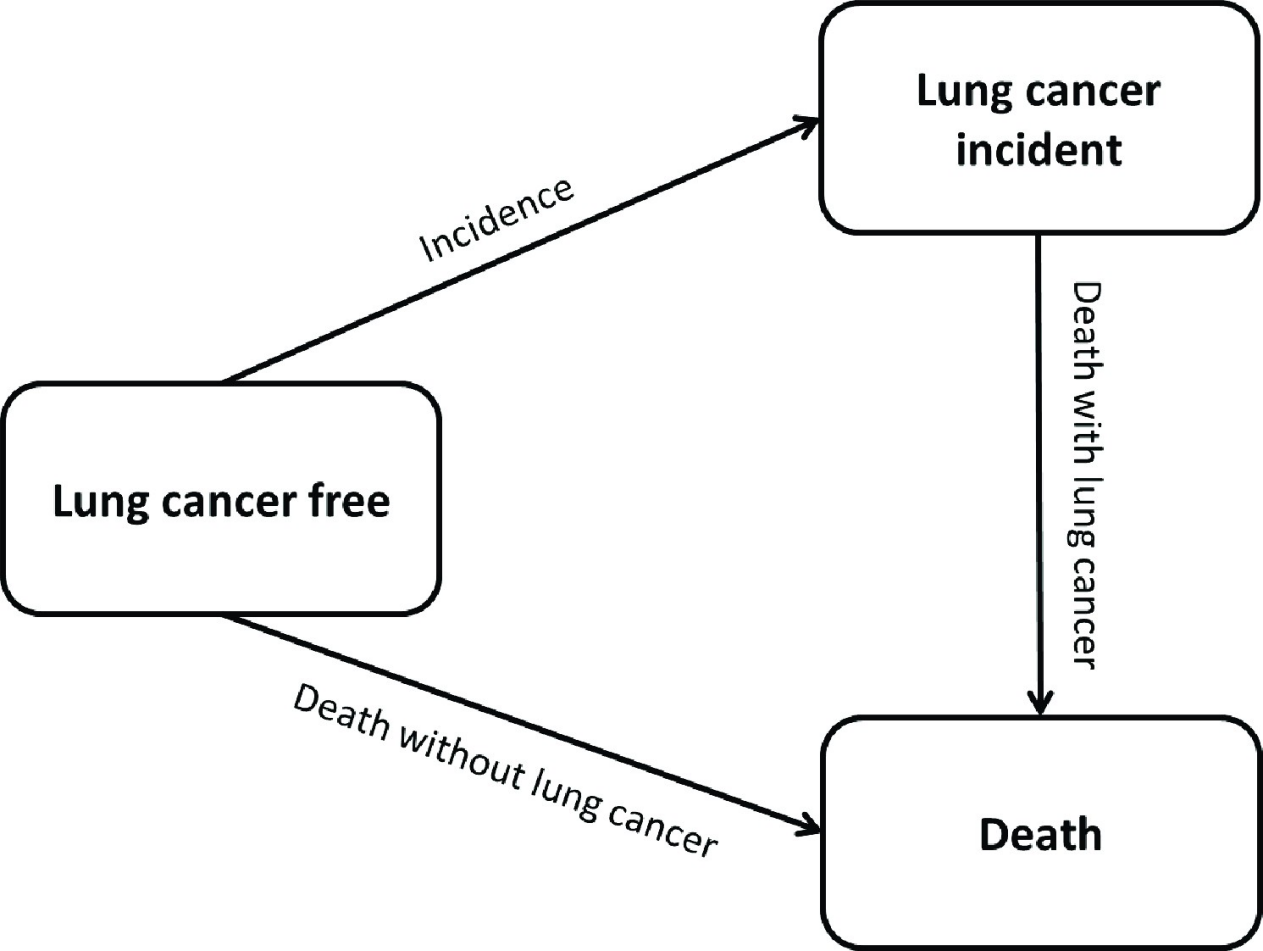
Three-state illness-death model (LC free, LC incident, and death) and the corresponding transitions (LC incidence, death without LC, and death with LC).

To gain a deeper insight into the dynamic mechanisms of inequalities in LC we analysed trends in gender- and income-specific LC incidence and mortality. Furthermore, we paid special attention to trends in inequalities in life years affected by LC and life years free of LC. To analyse these inequalities we used a multistate approach based on an illness-death model without recovery. The model defines two living states (LC free and LC incident), one death state and three possible transitions between the states (LC incidence, death with LC, and death without LC). Within this illness-death model, LC incidence and death without LC represent competing events (Fig 1).

Based on this illness-death model, proportional hazard survival models with constant baseline hazard were estimated separately for each type of transition, income group, and gender. Since LC incidence and death without LC represent competing events, cases with LC incidence were censored in the model death without LC at the exact time of LC incidence as they were no longer at risk for this transition (Fig 1, for more details on data preparation, see [40]). All models include a covariate for individual mean age in the respective period (in single-year age-groups as second-degree age polynomial). In order to analyse whether time trends in LC incidence and mortality risks differ between income groups, the data of the two time periods were combined into one model. Time trends were assessed by including time period as covariate in the models.

In a second step, the survival models were stratified by time period. From these models, smoothed age-specific rates for LC incidence, death with LC, and death without LC were predicted. The predicted transition rates were derived using the post-estimation command “predict” in STATA 14 [41].

The number of life years with and without LC was estimated using multistate life table analyses. The calculation of multistate life tables is largely based on matrix multiplication. For these analyses, we used the methodological approach described by Palloni [42]. To account for the interplay between incidence and mortality, age-specific hazard rates of all three transitions are needed to calculate the expected number of life years free of LC and affected by LC [42]. The number of life years with and without LC was calculated with multistate life tables in R 3.5.1 [43] using the predicted age-specific incidence and mortality rates as input. All confidence intervals are bootstrapped by performing 1000 replications.

## Results

In the two time periods, a total number of 2,511,790 individuals with 19,882 cases of LC incidence and 244,433 deaths (12,255 deaths with LC and 232,178 deaths without LC) were observed. Detailed information on the sex- and income-specific number of individuals, person-years of exposure, and number of incidence and death events can be found in Table 1.

**Table 1:** Characteristics of the study population aged 40 and older: number of insured individuals, exposures in person-years, and number of incident and death cases by type of transition, income group, time period, and gender.

			2006–2009		2014–2017	
	**Income group**		**Men**	**Women**	**Men**	**Women**
**LC incidence**	low	number of individuals	264,908	485,546	284,877	501,603
		person-years	921,316	1,740,619	983,645	1,793,461
		incident cases (%^1)^)	4,211 (1.59)	2,305 (0.47)	4,409 (1.55)	2,895 (0.58)
	higher	number of individuals	288,872	132,446	368,830	184,708
		person-years	1,041,619	478,576	1,337,328	661,389
		incident cases (%^1)^)	2,427 (0.84)	556 (0.42)	2,393 (0.65)	686 (0.37)
**Death with LC**	low	number of individuals	4,211	2,305	4,409	2,895
		person-years	7,936	4,314	8,366	5,565
		death cases (%^1)^)	2,755 (65.42)	1,350 (58.57)	2,948 (66.87)	1,674 (57.82)
	higher	number of individuals	2,427	556	2,393	686
		person-years	4,438	1,069	4,619	1,323
		death cases (%^1)^)	1,467 (60.44)	319 (57.37)	1,378 (57.58)	364 (53.06)
**Death without LC**	low	number of individuals	264,908	485,546	284,877	501,603
		person-years	921,315	1,740,619	983,646	1,793,462
		death cases (%^1)^)	34,435 (12.99)	58,960 (12.14)	39,718 (13.94)	56,596 (11.28)
	higher	number of individuals	288,872	132,446	368,830	184,708
		person-years	1,041,619	478,576	1,337,332	661,389
		death cases (%^1)^)	17,893 (6.19)	10,380 (7.84)	20,244 (5.49)	13,952 (7.55)

^1)^ Proportion of events (LC incidence, death with LC, death without LC) on the total number of individuals in the respective group.

### Time trends in inequalities in incidence and mortality risks

**Fig 2:**
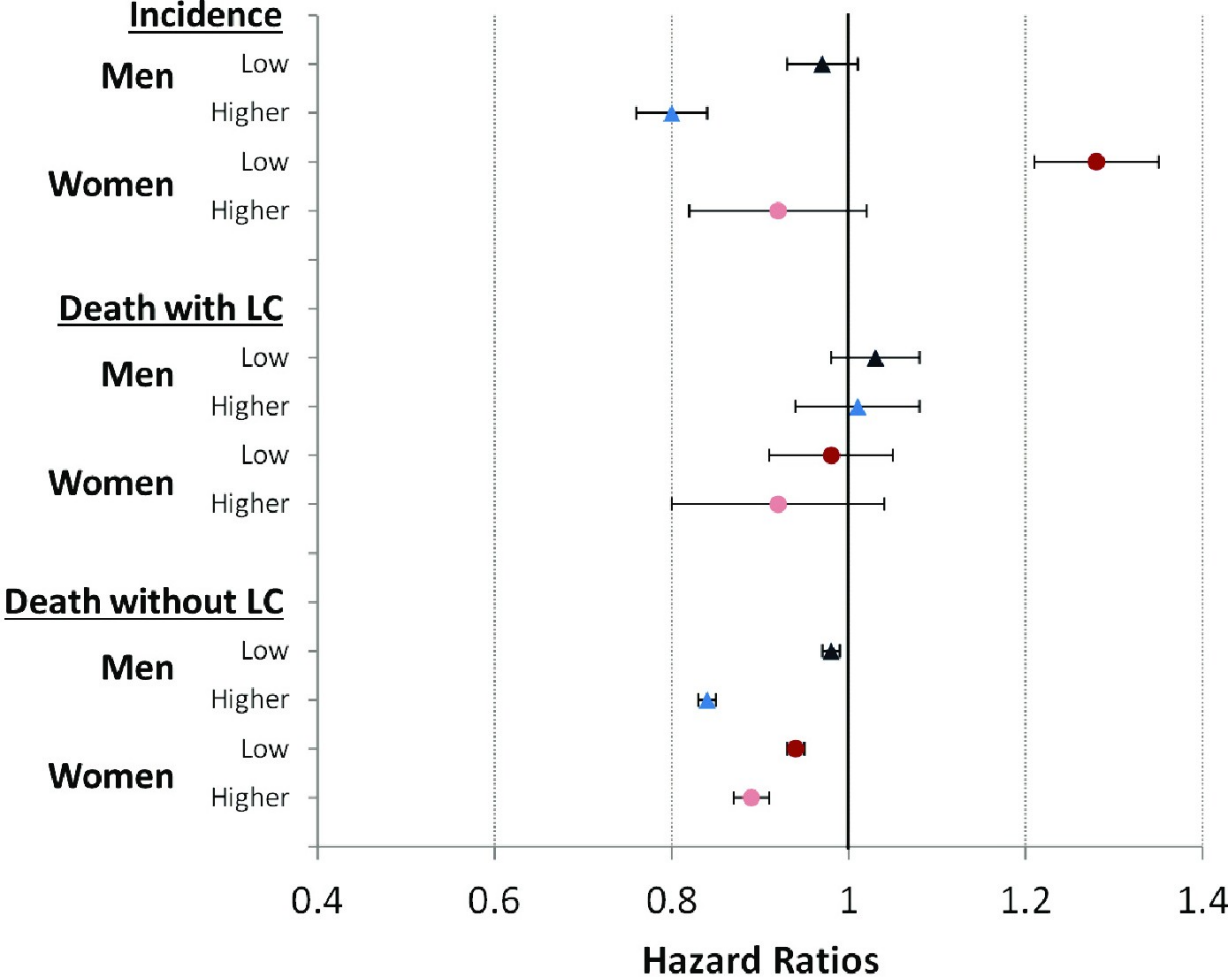
Time trends in risks of lung cancer incidence, death with lung cancer, and death without lung cancer by gender and income group (2014–2017 compared to 2006–2009 (Ref.)). Note: 95%-CI bootstrapped (with replacement) using 1000 replications; all analyses are controlled for age (in single-year age-groups as second-degree polynomial); LC lung cancer; Ref. Reference group.

In a first step, we estimated time trends in the risk of LC incidence, LC mortality and mortality without LC from proportional hazard models. The results are displayed in Fig 2. The analyses show that incidence risks in men decreased over time. With a reduction of about 20%, the decrease was much greater among men with higher incomes than in low-income men. For women, we found opposite trends. The analyses reveal that incidence risks among low-income women increased by more than 25%, while risks tend to decrease in women with higher incomes. While LC mortality risks in men remained largely stable over time, there might be a tendency towards decreasing mortality risks for women with higher incomes. However, the trends in mortality risks without LC are consistent and indicate declines in mortality for men and women. These declines were stronger in the higher than in the low-income group (Fig 2).

According to our illness-death model, we fitted proportional hazard models and predicted age-specific LC incidence rates, mortality rates after LC diagnosis, and mortality rates free of LC in a second step. The predicted rates fit the observed rates quite well although mortality rates after LC showed higher fluctuations as the number of deaths is limited in this group (see S1–S3 Figs).

### Time trends in inequalities in life years without LC and with LC

**Fig 3:**
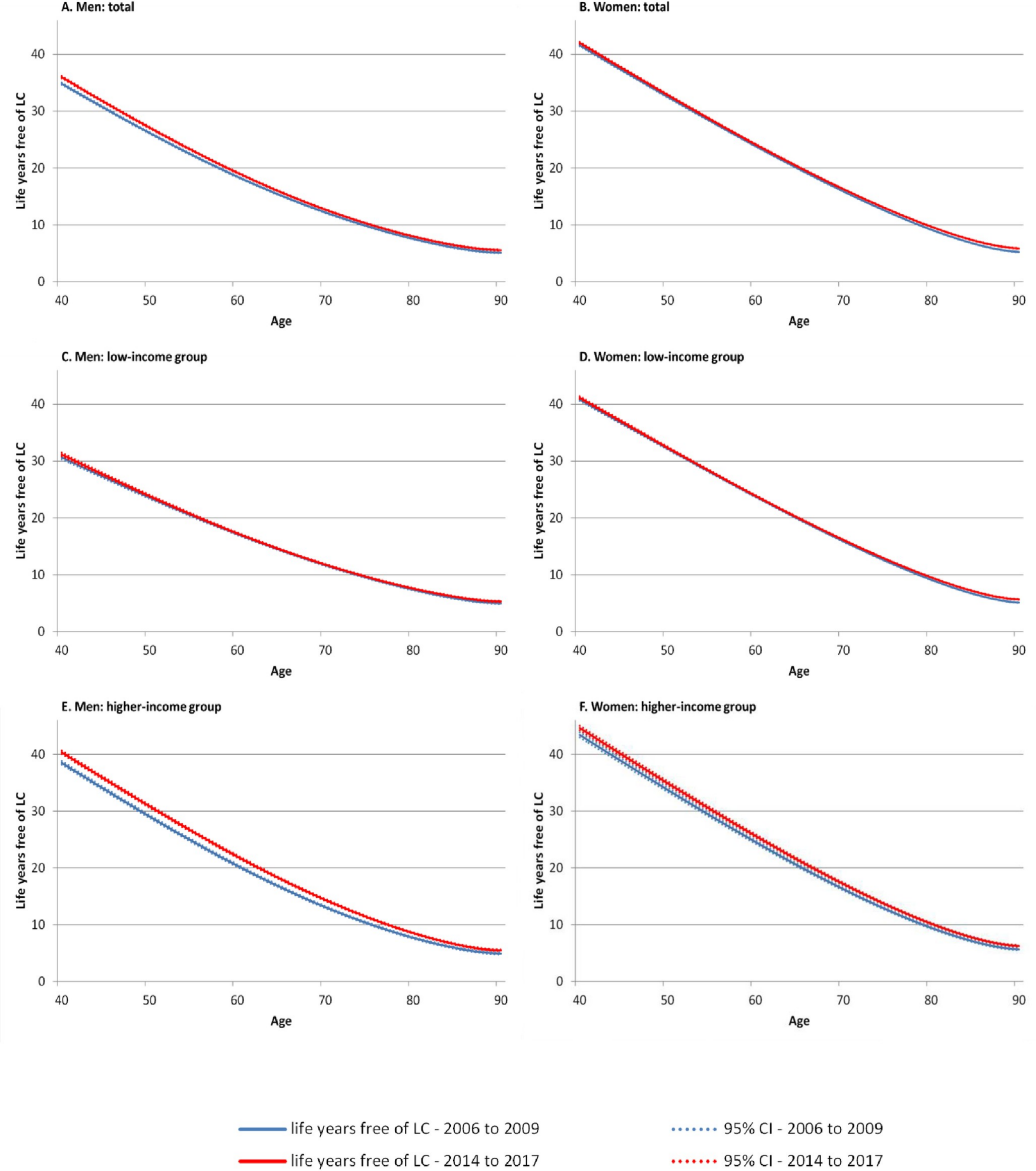
Time trends in remaining life years free of LC at age 40 and above by gender, period, and income group. Note: 95%-CI bootstrapped (with replacement) using 1000 replications, LC lung cancer.

Due to the substantial income inequalities in LC incidence and mortality (see S1–S3 Figs), the number of life years free of LC is lower among individuals having low incomes. This difference was most pronounced among men. Between the periods, life years free of LC increased in both gender. This development was mainly driven by the substantial gains in life years among men and women with higher incomes which can be found in nearly all ages. With respect to the low-income group, only slight increases were observed (Fig 3).

**Fig 4:**
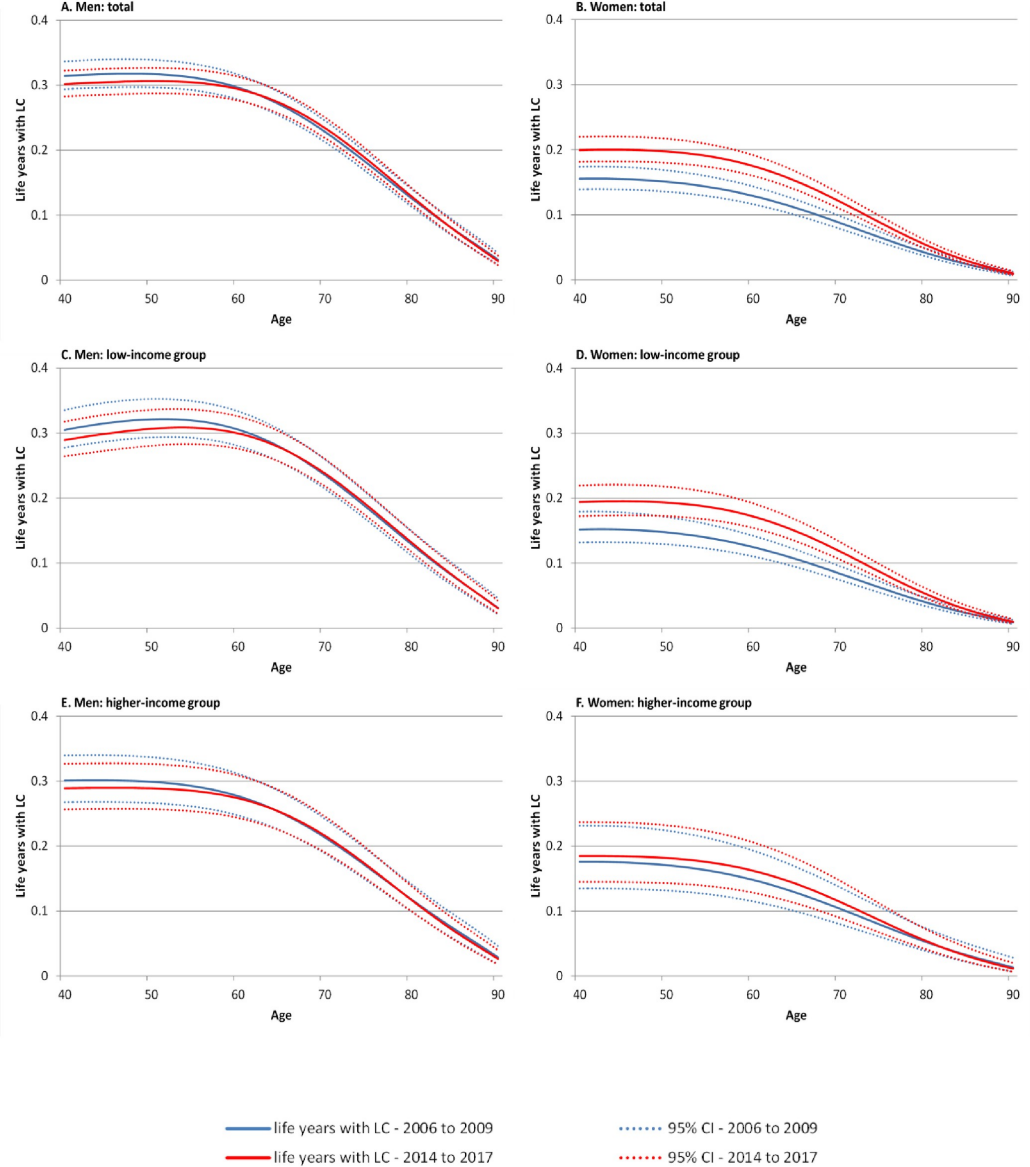
Time trends in remaining life years at age 40 and above with LC by gender, period, and income group. Note: 95%-CI bootstrapped (with replacement) using 1000 replications, LC lung cancer.

In men, trends in life years after LC incidence remained quite stable over time. However, below the age of 60 we found a slight trend towards decreasing life years affected by LC in total as well as in men with low and higher incomes. In contrast, an opposing trend was observed in women, where life years with LC increased clearly. This trend was particularly driven by the strong increase in the number of life years after LC incidence among women with low incomes (Fig 4).

**Fig 5:**
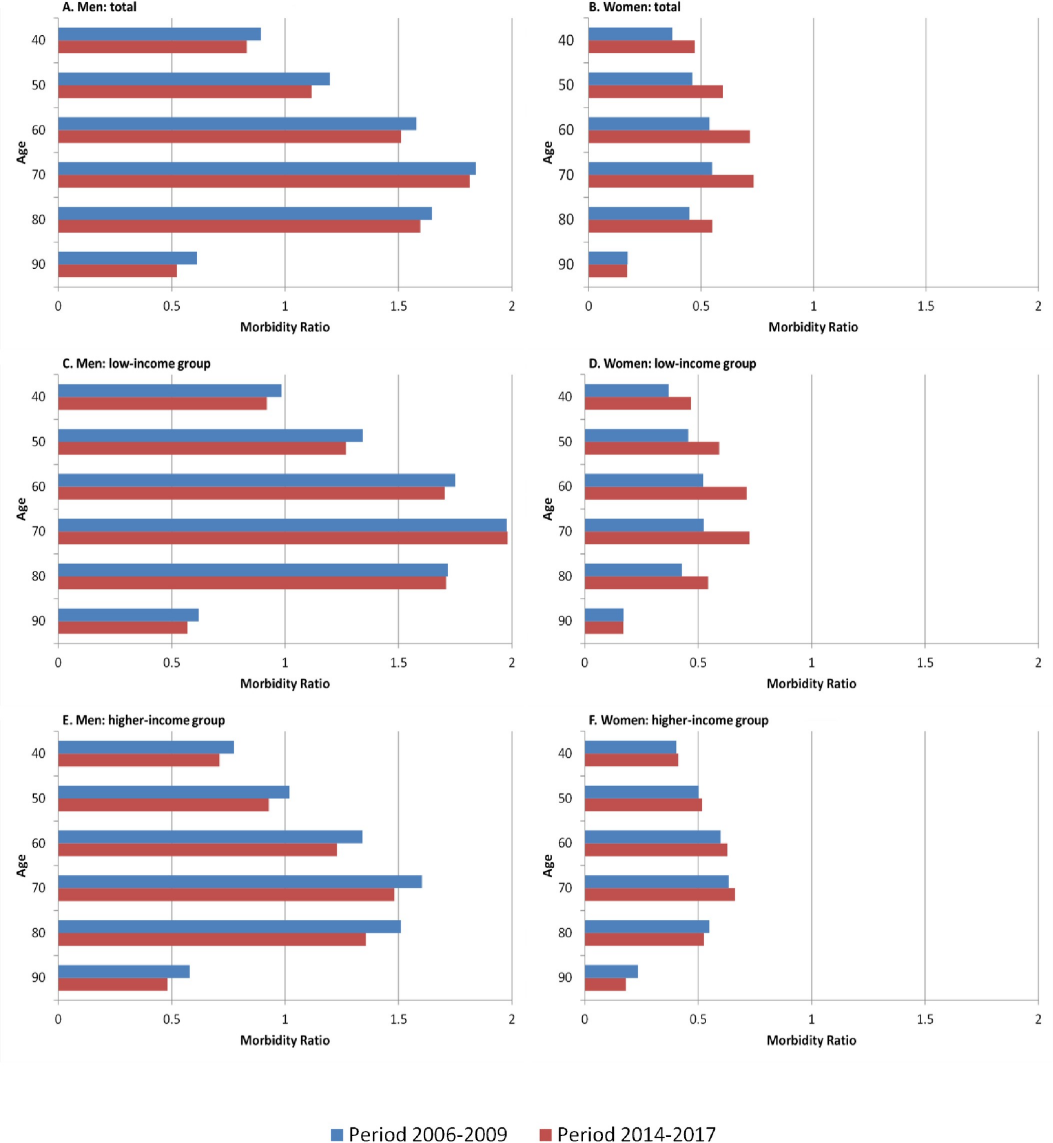
Time trends in proportion (in %) of life years with LC in total life years (Morbidity Ratio) by gender, period, income group, and age. Note: Morbidity Ratio = (life years with LC/(life years with LC + life years free of LC))*100, LC lung cancer.

To differentiate between relative and absolute compression or expansion, we calculated the morbidity ratio, which depicts changes in the proportion of life years with LC in total life expectancy. Between the periods, the proportion of life years affected by LC decreased in men but increased in women. While the reduction in men with low incomes is limited to age 60 and below, men with higher incomes benefitted over the full age range between 40 and 90 years. This finding indicates that life years free of LC increased at a faster pace than total life expectancy, leading to a relative compression among men irrespective of income. For women, however, the strong increase in the proportion of life years with LC in the low-income group is responsible for the increases in morbidity ratio of the total female population, leading to a relative expansion of LC among low-income women (Fig 5).

## Discussion

The aim of the study was to gain a deeper insight into the dynamic developments of gender- and income-specific inequalities in LC incidence and mortality over time and how these developments affected time trends in life years with and without LC in Germany. In particular, we wanted to investigate whether a compression or expansion in LC has taken place and whether time trends are socially patterned.

Our analyses showed that the incidence of LC decreased among men but increased among women (see S1 Fig). However, time trends differed with respect to income leading to increased inequalities in the length of life with and without LC over time. While life years free of LC increasing in both genders, this trend was mainly driven by individuals with higher incomes. Furthermore, life years affected by LC remained quite stable over time in men but increased in women. This increase in life years with LC in women is due to the strong growth in incidence rates in the low-income group over the last decade. The developments described above led to a relative compression among men, which, however, was more pronounced within the higher-income group due to stronger decreases in incidence rates over time. For women on the other hand, the increase in life years affected by LC in the low-income group led to a clear expansion in both absolute and relative terms while no substantial changes were found among women with higher incomes.

Our study is in line with the official cancer reports of the national public health institute [44], which also reported declining LC incidence rates in men but increasing rates in women. With respect to the reported time trends in life years free of LC and after morbidity onset changes in mortality after LC incidence played only a minor role since mortality rates remained largely stable over time. However, some studies report social inequalities in mortality after LC diagnosis [28,45,46], which were also evident for men and women below age 65 in our study (see S2 Fig). While previous research based on a linkage of German cancer registry data and information on the regional deprivation level reported SES inequalities in LC incidence for men but not for women [9], we also found substantial inequalities among the female population. This discrepancy can be explained by the shift in the income gradient in women over time which cannot be detected without conducting time trend analyses. While in the first period incidence rates in elderly women with higher incomes were higher than in the low-income group, this gradient turned in the second period as rates increased substantially in low-income women (see S1 Fig). This shift is mainly due to changes in rates at age 70 and above, i.e. the age range which accounts for the largest share of LC incidence cases. Therefore, our results suggest that focusing on social inequalities without addressing the development of inequalities over time could lead to a masking of inequalities in LC incidence, in particular among women. Moreover, it seems important to analyse changes over time also with respect to different age groups.

The most important explanation of trends in LC incidence lies in the development of risk factors over time, especially in smoking. Rising smoking rates among women in the 1960s and 1970s led to a convergence in smoking behaviour between genders and increased LC rates among women during the last decades substantially [21,22,27,32]. On the other hand, smoking prevention interventions and tobacco control policies implemented at the end of the 20th century reduced the high smoking rates among men [20,26] and fostered decreasing LC incidence rates in Germany. Progress in tobacco prevention was made and further prevention strategies have been proposed and discussed. However, tobacco control regulations remain weaker than in other European countries [2,47]. Although smoking prevalence is declining, this does not apply equally to all SES groups as smoking decreased more rapidly in men and women with high SES [27,48], which contributed to growing inequalities in LC.

Recent studies report substantial income inequalities in life expectancy. In particular, men with higher incomes are most advantaged since gains of additional life years over time were considerably higher than in men with low income, which led to increasing inequalities in life expectancy over time [49–51]. Since smoking-related diseases are among the main causes of gender and SES differences in mortality and life expectancy [31,49], further research is needed to understand the factors causing inequalities in smoking behaviour and to identify trends in health expectancy, particularly for smoking-related diseases.

### Strengths and limitations

This study is based on longitudinal data of large German health insurance provider covering a time period of 12 years. This dataset represents a complete insurance population and includes individual information on in- and outpatient diagnoses and income. Furthermore, individuals could be included in the analyses independent of their current health status. Hence, our findings are not influenced by health-related non-response, which occurs in survey data if participants drop out for health reasons [52]. Another strength of our study is that all information on diagnoses, mortality and SES needed for the analyses is included in one dataset and can be accessed at individual level. Analyses based on individual data are preferable to those at macro level because ecological fallacies, which might affect the interpretation of results, are avoided.

Furthermore the long observation period of 12 years, high case numbers, and the detailed individual information on LC incidence, mortality and income allowed us to conduct complex analyses on time trends in inequalities even if models were stratified by gender and income group. The data do not provide information on household income or household composition. Therefore, household income could not be used as SES indicator in our study. As the general income level among women is lower than that among men [53], using individual income information rather than household income may have affected the results. However, previous research has shown that, compared to other definitions of income (e.g. household income), individual income is an appropriate and robust SES indicator in studies investigating health inequalities [54]. In this study, the analyses on income inequalities in LC incidence and mortality are based on two income groups. Using more than two income groups would have provided a deeper insight into income inequalities in lung cancer incidence and mortality. However, since the number of incidence cases is limited within age, period, sex, and income groups, it was not possible to classify the population into more than two income groups.The analyses are based on a three-state illness-death model ignoring the possibility of recovery from LC as LC still represents one of the most fatal cancer diseases worldwide and the rate of 5-years survival is low (approximately 15% in men and 21% in women in Germany [44]). Moreover, research indicates that LC patients often suffer from several limitations, even after a successful surgery and therapy (e.g. loss of a lung wing, chemotherapy-induced polyneuropathy, psychological problems and fear of recurrence, cognitive impairment, and chronic fatigue) [55]. From a methodological perspective, considering recovery would require a post-observation period for at least five to ten years after the initial LC diagnosis (which had to be free of further LC diagnoses in recovered individuals) for all individuals who become LC incident within the two periods (2006–2009 and 2014–2017). Given that the database covers a total of 12 years, the analysis of time trends would have been severely limited. Therefore, we consider the used illness-death model without recovery to be most appropriate to investigate temporal trends in LC-associated health expectancies.

In terms of gender and age distributions the data are representative for the total population of Germany, but differ, however, in the composition of SES characteristics [38]. To address this issue, all analyses are controlled for or stratified by income. Therefore, our analyses remain unaffected by this limitation.

## Conclusion

Our study shows that the proportion of the average length of life spent with LC declined in men, leading to a relative compression in LC. For women on the other hand, the number of life years affected by LC increased over time which caused a clear expansion in women with low incomes. Moreover, the study reveals rising social inequalities in LC incidence as well as in life years with and without LC in both genders. In particular, men and women with lower incomes are disadvantaged since individuals with higher incomes experienced more favourable developments in life years free of LC and affected by LC over time.

Over the last decades there have been some effort in tobacco control (e.g. smoking ban in public places) and progress has been made to counteract the growing smoking epidemic in Germany. However, it is still important to strengthen efforts in primary prevention in order to reduce the smoking rate and thus smoking-related diseases in the German population. Policy makers and public health interventions should focus in particular on vulnerable groups to reduce the persisting social inequalities in LC.

## Supporting information

S1 Fig Observed and predicted values for lung cancer incidence rates for the periods 2006–2009 and 2014–2017 by gender and income group. (PDF)

S2 Fig Observed and predicted values for death rates after lung cancer incidence for the periods 2006–2009 and 2014–2017 by gender and income group. (PDF)

S3 Fig Observed and predicted values for death without lung cancer for the periods 2006–2009 and 2014–2017 by gender and income group. (PDF)
