# Supplementary material for: Compression, expansion, or maybe both? Growing inequalities in lung cancer in Germany
Source: PLoS One. 2020 Nov 20;15(11):e0242433. doi: 10.1371/journal.pone.0242433 (PMC7679006; doi:10.1371/journal.pone.0242433)

S1 Fig 2: Observed and predicted values for death rates after lung cancer incidence for the periods 2006-2009 and 2014-2017 by gender and income group

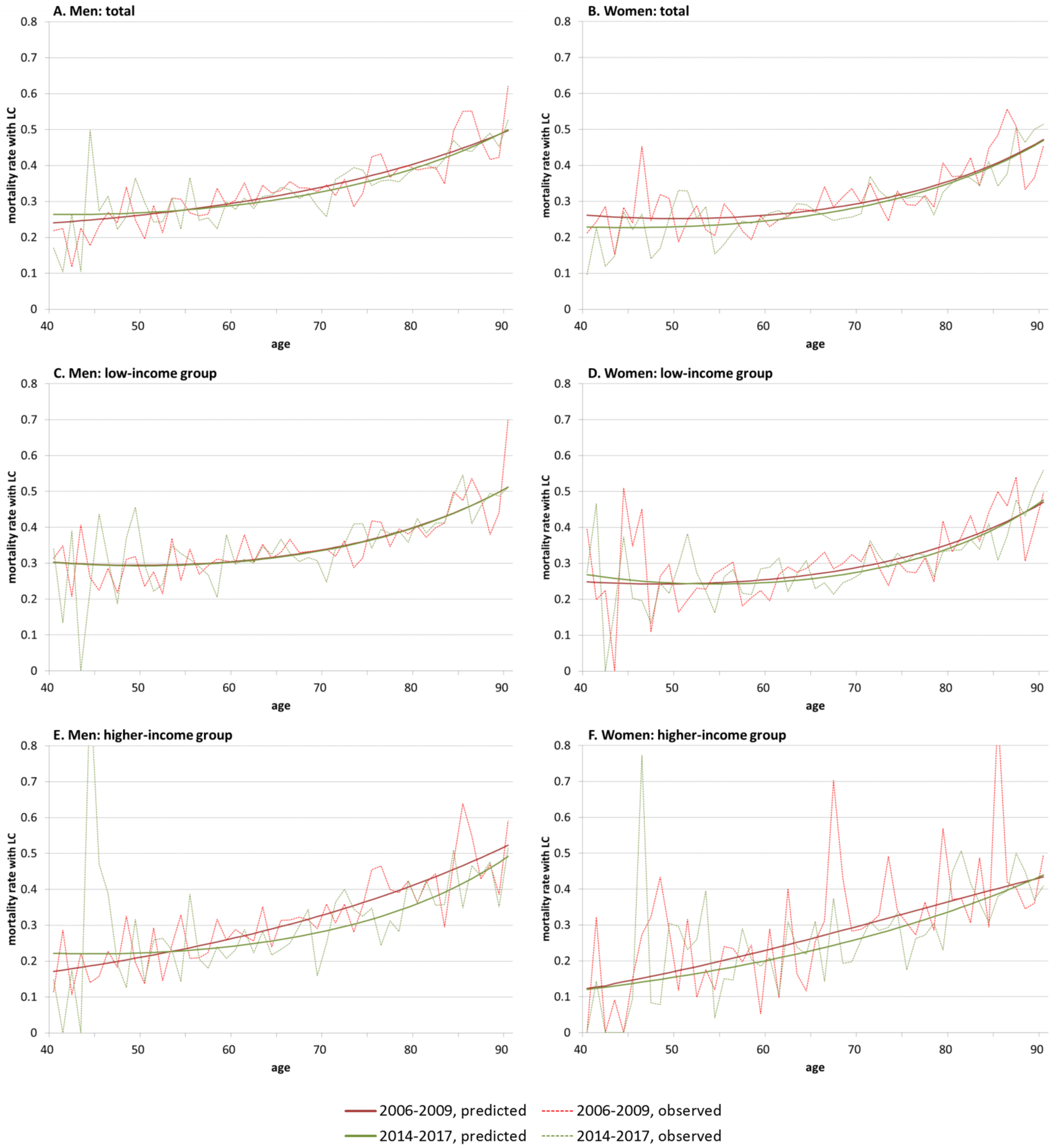

Supplement: S2 Fig — (PDF) [file pone.0242433.s002.pdf]
